# Stroke frequency, associated factors, and clinical features in primary systemic vasculitis: a multicentric observational study

**DOI:** 10.1007/s00415-024-12251-1

**Published:** 2024-03-12

**Authors:** Ruth Geraldes, Monica Santos, Cristina Ponte, Anthea Craven, Lillian Barra, Joanna C. Robson, Nevin Hammam, Jason Springer, Jöerg Henes, Alojzija Hocevar, Jukka Putaala, Ernestina Santos, Liza Rajasekhar, Thomas Daikeler, Omer Karadag, Andreia Costa, Nader Khalidi, Christian Pagnoux, Patrícia Canhão, Teresa Pinho e Melo, Ana Catarina Fonseca, José M. Ferro, João Eurico Fonseca, Ravi Suppiah, Richard A. Watts, Peter Grayson, Peter A. Merkel, Raashid A. Luqmani, the DCVAS Study Group

**Affiliations:** 1 Neurology Department, Wexham Park Hospital, Frimley Health Foundation Trust, Slough, UK; 2 Department of Clinical Neurosciences, Oxford University Hospitals, Oxford, UK; 3 Serviço de Neurologia, Departamento de Neurociências e Saúde Mental, Centro Hospitalar Universitário Lisboa Norte, Lisbon, Portugal; 4 Centro de Estudos Egas Moniz, Faculdade de Medicina da Universidade de Lisboa, Lisbon, Portugal; 5 Rheumatology and Metabolic Bone Diseases Department, Centro Hospitalar Universitário Lisboa Norte, Lisbon Academic Medical Centre, Lisbon, Portugal; 6 Rheumatology Research Unit, Instituto de Medicina Molecular, Faculdade de Medicina da Universidade de Lisboa, Lisbon Academic Medical Centre, Lisbon, Portugal; 7 Nuffield Department of Orthopaedics, Rheumatology and Musculoskeletal Sciences, Nuffield Orthopaedic Centre, Botnar Research Centre, University of Oxford, Oxford, UK; 8 Lawson Health Research Institute, London, ON Canada; 9 Centre for Health and Clinical Research, University of the West of England, Bristol, UK; 10 Rheumatology Department, Faculty of Medicine, Assiut University, Assiut, Egypt; 11 University of Kansas Medical Centre Institute, Lawrence, Kansas, KS USA; 12 Centre for Interdisciplinary Clinical Immunology, Rheumatology and Auto-Inflammatory Diseases, Department of Internal Medicine II (Oncology, Haematology, Immunology and Rheumatology), University Hospital Tuebingen, Tuebingen, Germany; 13 University Medical Centre Ljubljana, Ljubljana, Slovenia; 14 Helsinki University Central Hospital, Helsinki, Finland; 15 Hospital de Santo António, Centro Hospitalar Universitário do Porto, Porto, Portugal; 16 Unit for Multidisciplinary Research in Biomedicine, Instituto de Ciências Biomédicas de Abel Salazar, Universidade do Porto, Porto, Portugal; 17 NIMS, Hyderabad, India; 18 Department of Rheumatology and Clinical Research, University Hospital, Basel, Switzerland; 19 Division of Rheumatology, Department of Internal Medicine, Vasculitis Research Center, Hacettepe University School of Medicine, Ankara, Turkey; 20 Centro Hospitalar Universitário de São João, Porto, Portugal; 21 Neuroscience and Mental Health Department, Faculdade de Medicina da Universidade do Porto, Porto, Portugal; 22 Joseph’s Healthcare Hamilton and McMaster University, Hamilton, ON Canada; 23 Mount Sinai Hospital, Toronto, Canada; 24 Instituto de Medicina Molecular, Faculdade de Medicina da Universidade de Lisboa, Lisbon, Portugal; 25 Te Whatu Ora-Health, Wellington, New Zealand; 26 Norwich Medical School, Norwich, UK; 27 National Institutes of Health, NIAMS Vasculitis Translational Research Program, Bethesda, USA; 28 Division of Rheumatology, Department of Medicine, University of Pennsylvania, Philadelphia, USA; 29 Division of Epidemiology, Department of Biostatistics, Epidemiology, and Informatics, University of Pennsylvania, Philadelphia, USA

**Keywords:** Stroke, Primary systemic vasculitis, Transient ischaemic attack, Cerebrovascular event

## Abstract

**Objectives:**

The cerebral vessels may be affected in primary systemic vasculitis (PSV), but little is known about cerebrovascular events (CVEs) in this population. This study aimed to determine the frequency of CVEs at the time of diagnosis of PSV, to identify factors associated with CVEs in PSV, and to explore features and outcomes of stroke in patients with PSV.

**Methods:**

Data from adults newly diagnosed with PSV within the Diagnostic and Classification Criteria in VASculitis (DCVAS) study were analysed. Demographics, risk factors for vascular disease, and clinical features were compared between patients with PSV with and without CVE. Stroke subtypes and cumulative incidence of recurrent CVE during a prospective 6-month follow-up were also assessed.

**Results:**

The analysis included 4828 PSV patients, and a CVE was reported in 169 (3.50%, 95% CI 3.00–4.06): 102 (2.13% 95% CI 1.73–2.56) with stroke and 81 (1.68% 95% CI 1.33–2.08) with transient ischemic attack (TIA). The frequency of CVE was highest in Behçet’s disease (9.5%, 95% CI 5.79–14.37), polyarteritis nodosa (6.2%, 95% CI 3.25–10.61), and Takayasu’s arteritis (6.0%, 95% CI 4.30–8.19), and lowest in microscopic polyangiitis (2.2%, 95% CI 1.09–3.86), granulomatosis with polyangiitis (2.0%, 95% CI 1.20–3.01), cryoglobulinaemic vasculitis (1.9%, 95% CI 0.05–9.89), and IgA-vasculitis (Henoch-Schönlein) (0.4%, 95% CI 0.01–2.05). PSV patients had a 11.9% cumulative incidence of recurrent CVE during a 6-month follow-up period.

**Conclusion:**

CVEs affect a significant proportion of patients at time of PSV diagnosis, and the frequency varies widely among different vasculitis, being higher in Behçet’s. Overall, CVE in PSV is not explained by traditional vascular risk factors and has a high risk of CVE recurrence.

**Supplementary Information:**

The online version contains supplementary material available at 10.1007/s00415-024-12251-1.

## Introduction

Primary systemic vasculitis (PSV) comprises a heterogeneous group of inflammatory diseases affecting large, medium, and/or small vessels in different organs, including extra- and intra-cranial involvement. Overall, vasculitis is a rare cause of stroke, but its frequency in patients with stroke varies globally [1, 2]. The epidemiologic data may be dependent on the local experience and evaluation strategies used for diagnosing rare causes of stroke, such as PSV, even in young people where the proportion of stroke due to vasculitis is thought to be higher [3, 4]. Moreover, people with PSV seem to have a greater risk of cerebrovascular disease when compared to the general population, particularly those with Takayasu’s arteritis (TAK) [5], giant cell arteritis (GCA) [6, 7], anti-neutrophil cytoplasmic antibody (ANCA)-associated vasculitis [8–10], or Behçet’s disease [11]. This increased risk may relate to direct involvement of vasculitis in the central nervous system (CNS) [12, 13], extracranial organ involvement (e.g. heart) [14], accelerated rates of atherosclerosis [15], or potential side effect of treatments for PSV. Stroke early in disease course is perhaps more likely directly related to vasculitis than to atherosclerosis, other vascular or cardiac conditions, or treatment complications, which may tend to accrue and be more significant later in PSV disease course.

However, little is known about the frequency and features of stroke at PSV onset. The frequency of stroke in PSV has been studied in relatively small and heterogeneous cohorts, most commonly retrospectively assessed over the whole disease course [10, 16–25]). Ischaemic stroke has been described as the most common subtype of stroke in these patients [16, 22], but there are limited available data on the frequency of haemorrhagic stroke and cerebral venous thrombosis (CVT) in PSV [26–30], except for CVT in Behçet’s disease [31].

This study aimed to determine the frequency and subtypes of stroke and transient ischaemic attack (TIA) at the time of diagnosis of PSV and to identify features associated with occurrence of cerebrovascular events (CVEs) in PSV. In addition, recurrence of stroke/TIA and stroke-related disability were explored in patients with PSV and compared to patients without PSV.

## Methods

### Study design and patients

This investigation was a multinational observational sub-study of the Diagnostic and Classification Criteria in VASculitis (DCVAS) study, which has been described in detail elsewhere [32]. Consecutive adult patients where PSV was a potential diagnosis for current illness were recruited from academic and community practices, between January 2011 and December 2017. All cases were enrolled within 2 years of diagnosis, except for patients with TAK and PAN who were enrolled up to 5 years after diagnosis to increase recruitment chances. Patients were prospectively followed for 6 months after enrolment date.

From the DCVAS database, data were extracted on patients with a diagnosis of PSV confirmed by the submitting physician at 6-months, as per the DCVAS study protocol [32] including large vessel-vessel (GCA, TAK), medium-vessel (polyarteritis nodosa (PAN)), small-vessel [eosinophilic granulomatosis with polyangiitis (EGPA), granulomatosis with polyangiitis (GPA), microscopic angiitis (MPA)], Behçet’s disease, and other forms of vasculitis. Data were also extracted on patients with a diagnosis of other autoimmune/systemic illnesses with a similar presentation to PSV (“PSV mimics”), which was used as a comparator group. Patients with single-organ vasculitis, including primary angiitis of the central nervous system, or diagnosed with two forms of vasculitis were excluded. Data from all PSV cases were analysed cross-sectionally.

All DCVAS sites were invited and agreed a priori to participate in the pre-specified Stroke sub-study (listed in Online Resource 1). The Stroke sub-study included patients experiencing any CVE from symptom onset until a vasculitis or a vasculitis mimic diagnosis and consisted in retrospective collection of data at enrolment date, and prospective data collection during the 6-month follow-up period in the main study. Based on the literature, assuming a combined 7% frequency of CVE in PSV [6–11, 16, 17, 20–23], a minimum sample of 140 stroke patients was calculated.

### Primary, secondary, and exploratory objectives

The primary aim of this study was to determine the frequency of CVE in patients with PSV (question 1). Secondary aims were to analyse factors associated with CVE occurrence in PSV (question 2); and to assess the different characteristics of stroke subtypes, disability, and 6-month recurrence rate in PSV patients (question 3). Exploratory aims included comparing patients with CVE and PSV with “PSV mimics” (question 4). Data from all PSV cases included in the DCVAS were analysed cross-sectionally to answer questions 1 and 2. Cross-sectional and prospective data from the Stroke sub-study were used to answer questions 3 and 4.

### Data collection and definitions

Organ involvement, including CVE, laboratory and imaging findings, present from first symptom onset to diagnosis of PSV/PSV mimics, were recorded in the DCVAS study [33]. Dates of organ involvement manifestations, including CVE occurrence dates, were not available. The stroke sub-study case report form (CRF) included additional data elements on CVE (stroke and TIA) during the above-mentioned time periods, including diagnosis and aetiological investigations (Online Resource 5) made by each centre investigator, as per local standard of care. Clinical definitions were used for ischaemic stroke, TIA, haemorrhagic stroke [intracerebral haemorrhage and subarachnoid haemorrhage (SAH)], and cerebral venous thrombosis and supported by imaging data [34, 35]. Aetiology of ischaemic CVE was defined according to ‘Trial of ORG 10172 in acute stroke treatment’ (TOAST) classification categories [36]. Causes for intracerebral haemorrhage were noted separately (small-vessel disease, vascular malformation, amyloid angiopathy, haematological disorders, cryptogenic). Asymptomatic ischaemic or haemorrhagic cerebral lesions were not considered to be CVE. The arterial territory involved in ischaemic events was classified combining the clinical presentation [37] and confirmed by the reported imaging. Stroke data documented in the CRFs were reviewed case by case by two neurologists (RG, MS). The diagnosis, aetiology and location of strokes were agreed upon by consensus after full review of all data. The original brain imaging scans were not available for review.

Comorbidities prior to the symptom onset of the current illness (PSV or mimics), including history of CVE (‘Previous CVE’), and the Vasculitis Damage Index (VDI) [38] at 6 months were captured. In the stroke sub-study, cumulative incidence of recurrent CVE during the 6-month period of follow-up was determined. Stroke disability at 6-month follow-up was assessed with the modified Rankin scale (mRankin) [39].

### Statistical analysis

Descriptive analyses were conducted on the frequency and characteristics of CVEs in PSV (all) and each individual forms of PSV. Continuous variables were expressed as means (standard deviations), or medians (interquartile range). Categorical variables were expressed as absolute numbers and percentages.

Patients with PSV and CVE were compared to patients with PSV without CVE regarding features potentially associated with the occurrence of CVE (comorbidities, organ involvement, presence of new onset headache or peripheral neuropathy). Univariate analysis included Pearson chi-square/Fisher exact test, *t* Student test, or Mann–Whitney test, as appropriate. Age, disease duration, ethnicity, sex and variables that were significant in the univariate analysis were included in a binary logistic regression model used to identify factors associated with CVE for the whole PSV group. Results are summarized as odds ratios (OR) and 95% confidence intervals (CI). Finally, descriptive statistics on stroke were calculated for PSV mimics and patients in the Stroke-Biobank, as done for patients with PSV. Two-sided *p*-values were considered statistically significant when < 0.05. Statistical analyses were performed using SPSS Statistic Editor version 28.

### Standard protocol approvals, registrations, and patient consents

The study was approved by the Berkshire Research Ethics Committee (10/H505/19). The DCVAS study is listed in the ClinicalTrials.gov database (NCT01066208). All sites obtained any additional ethical and institutional approvals required for their jurisdiction. The DCVAS study is in accordance with the 1964 Declaration of Helsinki and ethical approval was obtained by national and local ethics committees in accordance with national legislation. All patients signed an informed consent form.

### Data availability

Data from the DCVAS study used for this study analyses are available from the DCVAS Steering Committee on reasonable request.

## Results

**Fig. 1:**
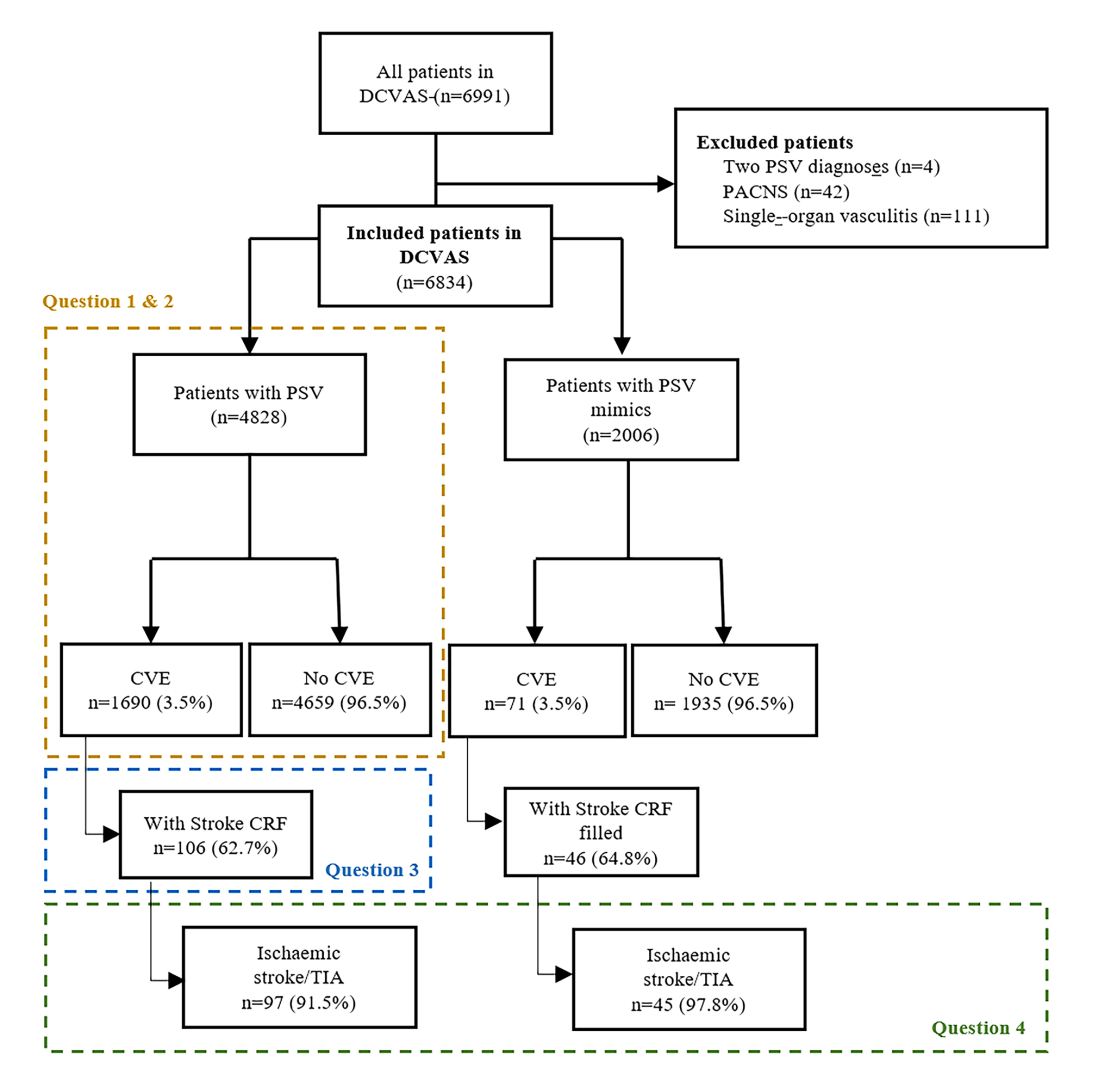
Flowchart of patients included in the study. *IMM* Instituto de Medicina Molecular, DCVAS—Diagnostic & Classification Criteria in Vasculitis study; *PSV* primary systemic vasculitis; *PACNS* primary angiitis of the central nervous system; *CVE* cerebrovascular event; *CRF* case report form; *TIA* transient ischaemic attack

From the initial 6991 patients with data submitted into the DCVAS study, 6834 patients were included (4828 with PSV and 2006 with PSV mimics) (Fig. 1). Patients with PSV had a median age of 61.0 (IQR 30.0) years, 59.4% were female, and the median disease duration was 10 (IQR 10.0) months (5.9% with no exact disease duration available) (Table 1).

**Table 1:** Comparison of patients with primary systemic vasculitis with and without cerebrovascular events regarding baseline characteristics, systemic organ involvement and cardiovascular risk factors

	With CVE (*n* = 169)	Without CVE (*n* = 4659)	*p*-Value
Median age (IQR)	59.0 (35.0)	61.0 (30.0)	0.37
Median disease duration (IQR) (months)	11.0 (11.0)	10.0 (10.0)	0.03
Female sex (%)	98 (58.0)	2770 (59.5)	0.70
Ethnicity			
White (%)	112 (66.3)	3357 (72.1)	0.10
Black/African (%)	5 (3.0)	61 (1.3)	0.08
Asian (%)	32 (18.9)	822 (17.6)	0.67
Middle East (%)	23 (13.6)	357 (7.7)	0.005
Latin (%)	1 (0.6)	97 (2.1)	0.134
Systemic involvement			
Skin (%)	54 (32.0)	1464 (31.4)	0.88
Cardiovascular (%)	56 (33.1)	1108 (23.8)	0.005
Musculoskeletal (%)	83 (49.1)	2451 (52.6)	0.37
Chest/pulmonary (%)	59 (34.9)	1861 (39.9)	0.19
Gastrointestinal (%)	51 (30.2)	1076 (23.1)	0.03
Eye (%)	78 (46.2)	1309 (28.1)	< 0.001
Gynaecologic/urological (%)	27 (16.0)	525 (11.3)	0.06
Ear, nose, and throat (%)	54 (32.0)	2010 (43.1)	0.04
Median total VDI (IQR)	83 (49.1)	2451 (52.6)	0.37
Other neurological symptoms			
New headache (%)	57 (33.7)	1152 (24.7)	0.008
Neuropathy (%)	14 (8.3)	357 (7.7)	0.776
Cardiovascular risk factors			
Hypertension (%)	57 (33.7)	1370 (29.4)	0.23
Diabetes (%)	20 (11.8)	391 (8.4)	0.112
Dyslipidaemia (%)	27 (16.0)	553 (11.9)	0.11
Ever smoker (%)	66 (40.0)	1739 (38.8)	0.76
Coronary heart disease (%)	5 (3.0)	250 (5.4)	0.11
Heart failure (%)	1 (0.6)	76 (1.6)	0.24
Peripheral vascular disease (%)	3 (1.8)	86 (1.8)	0.62
Previous CVECVE (%)	15 (8.9)	86 (1.8)	< 0.001
Malignancy (%)	17 (10.1)	273 (5.9)	0.02

*CVE* cerebrovascular event; *previous CVE* previous history of CVE; *PSV* primary systemic vasculitis; *IQR* intra-quartile range; *VDI* vasculitis damage index

### CVE frequencies differed between PSVs

A total of 183 CVEs (any type) occurred in 169 (3.50%, 95% CI 3.00–4.06) patients diagnosed with PSV. Specifically, 102 (2.13% 95% CI 1.73–2.56) were stroke and 81 (1.68% 95% CI 1.33, 2.08) were TIA (Table 2). The frequency of CVE was highest in Behçet’s disease (9.5%, 95% CI 5.79–14.37), PAN (6.2%, 95% CI 3.25–10.61), and TAK (6.0%, 95% CI 4.30–8.19), and lowest in GCA (3.6%, 95% CI 2.59–4.77), EGPA (2.9%, 95% CI 1.45–5.09), MPA (2.2%, 95% CI 1.09–3.86), GPA (2.0%, 95% CI 1.20–3.01), cryoglobulinaemic vasculitis (1.9%, 95% CI 0.05–9.89) and IgA vasculitis (Henoch-Schönlein, (0.4%, 95% CI 0.01–2.05).

**Table 2:** Frequency of cerebrovascular events after onset of primary systemic vasculitis

	Any CVE *N* (%)	TIA *N* (%)	Stroke *N* (%)	Total *N* (%)
All primary systemic vasculitis	169 (3.5)	81 (1.7)	102 (2.2)	4828 (100.0)
Large-vessel vasculitis				
Takayasu’s arteritis	38 (6.0)	19 (3.0)	22 (3.5)	630 (13.0)
Giant cell arteritis	43 (3.6)	18 (1.5)	28 (2.3)	1206 (25.0)
Isolated aortitis	1 (2.9)	1 (2.9)	0 (0)	35 (0.7)
Other large-vessel vasculitis	4 (4.7)	3 (3.5)	2 (2.4)	85 (1.8)
Medium-vessel vasculitis				
Polyarteritis nodosa	12 (6.2)	4 (2.1)	8 (4.1)	193 (4.0)
Small-vessel vasculitis				
Granulomatosis with polyangiitis	20 (2.0)	10 (1.0)	13 (1.3)	1021 (21.1)
Eosinophilic granulomatosis with polyangiitis	11 (2.9)	3 (0.8)	8 (2.1)	382 (7.9)
Microscopic polyangiitis	11 (2.2)	6 (1.2)	8 (1.6)	505 (10.5)
Other small-vessel vasculitis	5 (2.6)	3 (1.6)	2 (1.1)	189 (3.9)
IgA vasculitis (Henoch-Schönlein)	1 (0.4)	1 (0.4)	0 (0)	270 (5.6)
Cryoglobulinaemic vasculitis	1 (1.9)	1 (1.9)	1 (1.9)	54 (1.1)
Other				
Behçet’s disease	19 (9.5)	12 (6.0)	7 (3.5)	201 (4.2)
Other with no specific vessel size	3 (5.3)	0 (0)	3 (5.3)	57 (1.2)

*CVE* cerebrovascular event; *TIA* transient ischemic attack

### Vasculitis and non-vasculitis related factors influence the occurrence of CVE

Univariate analyses comparing patients with PSV with and without CVE regarding baseline characteristics, systemic organ involvement, comorbidities/cardiovascular risk factors are summarized in Table 1. In the multivariate analysis (Online Resource 2), patients with Previous CVE (OR 4.09, 95% CI 2.11–7.92, *p* < 0.001) and malignancy (OR 2.51, 95% CI 1.29–3.92, *p* = 0.004) were more likely to experience a CVE. Eye (OR 2.37, 95% CI 1.67–3.37, *p* < 0.001), cardiovascular (OR 1.56, 95% CI 1.08–2.25, *p* = 0.02), and gastrointestinal (OR 1.54, 95% CI 1.05–2.28, *p* = 0.03) involvement of vasculitis were each associated with a higher risk of CVE, while PSV with ear-nose-throat involvement (OR 0.59, 95% CI 0.41–0.86, *p* = 0.006) were less likely to experience a CVE. Presence of a new headache (OR 1.65, 95% CI 1.12–2.44, *p* = 0.01) was also more common in PSV with CVE. Traditional cardiovascular risk factors (arterial hypertension, diabetes, dyslipidaemia) were not associated with increased risk of CVE.

Data on inflammatory parameters were available in 69.8% of patients with PSV, and no differences were found between patients with or without CVE in median maximum values of serum C reactive protein (CRP) (36.5 [IQR 107.0] vs 46.6 [IQR 94.8 mg/L, *p* = 0.38) or erythrocyte sedimentation rate (52.5 [IQR 47.0] vs 59.0 [IQR 56.0] mm/hr, *p* = 0.30).

Description of demographic characteristics of each PSV is displayed in Online Resource 3. The occurrence of CVE was associated with the presence of new-onset headache (36.8% vs 16.5%, *p* = 0.04) and eye involvement (78.9% vs 53.8%, *p* = 0.03) in the Behçet’s subgroup, while in the PAN subgroup, only gastrointestinal involvement was associated with the occurrence of CVE (75.0% vs 43.6%, *p* = 0.03). Skin (21.1% vs 6.9%, *p* = 0.006) and eye (44.7% vs 13.9%, *p* < 0.001) involvement was more common and median VDI scores were higher (3.0 [IQR 4.0] vs 1.0 [IQR 2.0], *p* < 0.001) in patients with TAK with CVE compared to those without CVE. Hypertension was more common in patients with GCA and CVE (62.8% vs 45.7%, *p* = 0.03), CVE did not associate with any other organ involvement in GCA. Previous CVE (14.0% vs 3.3%, *p* = 0.004) was more common in TAK (7.9% vs 0.8%, *p* = 0.009) and GCA (14.0% vs 3.3%, *p* = 0.004) patients with CVE, compared to patients without.

In the patients with small-vessel PSV, the occurrence of CVE was associated with older median age (66.0 [IQR 8.0] vs 59.0 [23.0], *p* = 0.04), higher median CRP (115.0 [IQR 137.0] vs 55.0 [IQR 113.28] mg/L, *p* = 0.02), higher median VDI (3.0 [IQR 3.0] vs 2.0 [IQR 2.0], *p* < 0.001), hypertension (45.2% vs 26.1%, *p* = 0.005), Previous CVE (11.9% vs 1.7%, *p* < 0.001), and malignancy (14.3% vs 5.4%, *p* = 0.03). In the subgroup of patients with ANCA-positive EGPA, GPA, and MPA (*n* = 1561), similar results were observed except for malignancy, which was no longer significant (11.8% vs 5.6% *p* = 0.13).

### In PSV most CVEs are ischemic and have a significant recurrence rate at 6-months

The Stroke sub-study CRF was filled out in 106 of 169 (63%) cases of PSV with reported CVE in the DCVAS database. Brain imaging reports were available in 96.2% of CRFs. Compared to cases with CVE without available data (*n* = 63), cases of CVE with available data in the Stroke CRF (*n* = 106) were older (median age 64.0 [IQR 33.0] vs 51.0 [IQR 35.0] years, *p* = 0.05), more frequently White (73.6% vs 54.0%, *p* = 0.009), less likely Asian (12.3% vs 30.2%, *p* = 0.004), and more frequently ever-smokers (47.1% vs 27.9%, *p* = 0.02). Diagnoses were also more frequently GCA (31.8% vs 14.3%, *p* = 0.008) and EGPA (9.3% vs 1.6%, *p* = 0.041) and less frequently TA (15.9% vs 33.3%, *p* = 0.008).

**Fig. 2:**
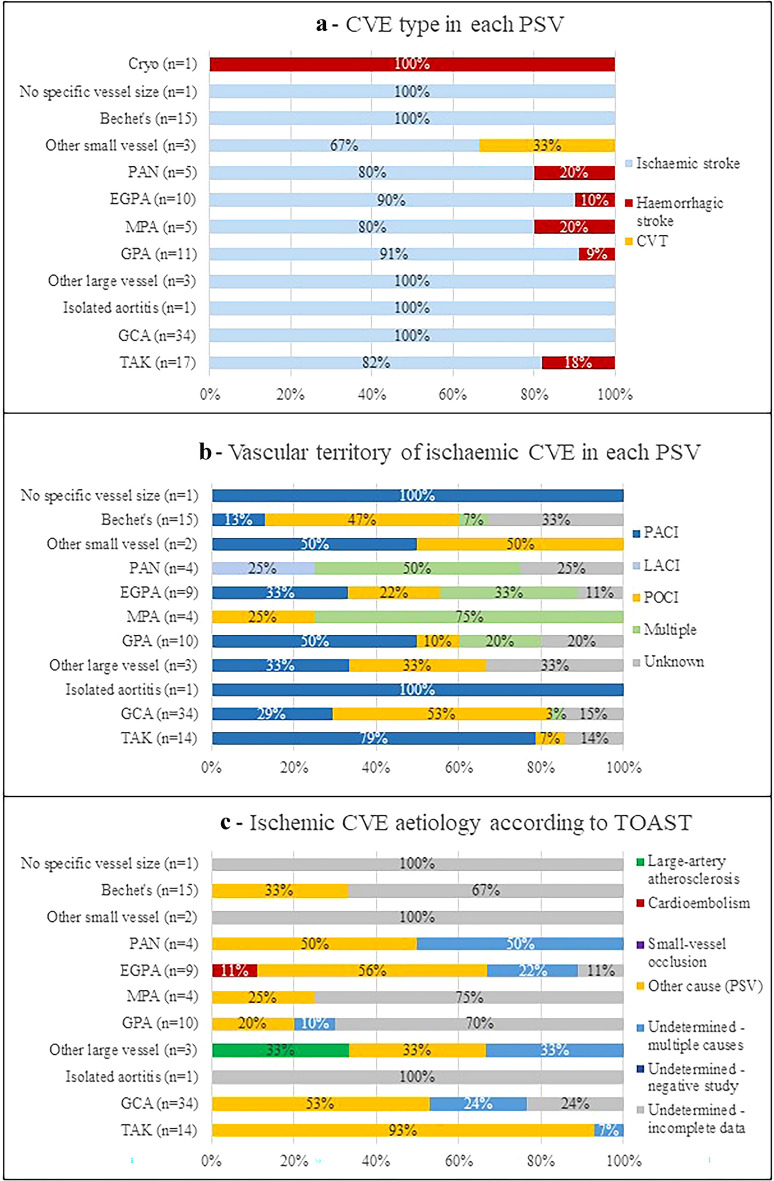
Characteristics of cerebral vascular events in each form of primary systemic vasculitis. **a** Type of cerebral vasculitis events. Ischaemic CVE was more common than haemorrhagic stroke in most PSVs. **b** Vascular territory of involvement in ischaemic cerebral vasculitis events. **c** Aetiology of ischaemic cerebral vasculitis events. CVE was attributed to vasculitis and classified in ‘Other determined cause’ in several of the PSVs. In EGPA, two CVE were attributed to cardioembolism, one associated with vasculitis-related cardiac ulceration, and one associated to a non-PSV related cardiomyopathy. The cause of CVE was not possible to ascertain in 32% of all PSVs. *CVE* cerebral vasculitis events; *Cryo* cryoglobulinaemic vasculitis; *PAN* polyarteritis nodosa; *EGPA* eosinophilic granulomatosis with polyangiitis; *MPA* microscopic polyangiitis; *GPA* granulomatosis with polyangiitis; *GCA* giant cell arteritis; *TAK* Takayasu’s arteritis; *CVT* cerebral venous thrombosis; *TACI* total anterior circular infarction; *PACI* partial anterior circular infarction; *LACI* lacunar infarction; *POCI* posterior circulation infarction; *PSV* primary systemic vasculitis

Most CVEs were ischaemic (*n* = 97; 91.5%), of which 49 (50.5%) TIA, and eight (7.5%) were haemorrhagic (parenchymal haematomas: 3 in TAK, 1 in EGPA, 1 in MPA; subarachnoid haemorrhage: 1 in GPA, 1 in cryoglobulinaemia; and 1 parenchymal haematoma with simultaneous subarachnoid haemorrhage in PAN). One (0.9%) cerebral venous thrombosis (CVT) was reported in a patient with ‘other small vessel PSV’ diagnosis (Fig. 2a).

Regarding ischaemic CVEs, anterior circulation ischaemia was present in 35 (36.1%) patients (particularly those with TAK), posterior circulation in 32 (32.7%) (especially in GCA), and multiple territories in 12 (12.2%) (distinctly in MPA, PAN and EGPA) (Fig. 2b). About half of ischaemic strokes (*n* = 47; 48.5%) were classified as ‘other determined aetiology’, all of which were attributed to PSV (Fig. 2c). Additionally, PSV was also one of the aetiologies in 11 of 15 patients with stroke attributed to ‘two or more causes’. Other causes of CVE identified simultaneously with PSV included cardioembolism (*n* = 5), large-vessel atherosclerosis (*n* = 2), small-vessel occlusion (*n* = 2), and infection (herpes encephalitis) (*n* = 1).

The cause of haemorrhagic CVE was at least partially attributed to PSV in three cases and, in four cases, to non-inflammatory cerebral small-vessel disease. No vascular malformations were described in patients with SAH. Three cases of asymptomatic cerebral aneurysms were reported: 2 in patients with related ischaemic stroke (GCA and Behçet’s), and 1 in a patient with TAK and hypertensive parenchymal haemorrhage (PH).

Information on treatment of ischaemic CVE was available in 92 cases: 63 (68.5%) cases were given aspirin and 8 (8.7%) two antiplatelet drugs. Eleven cases (12.0%) were given heparin and 10 (11.0%) oral anticoagulation. Regarding acute treatment of ischaemic stroke, only one case was treated with recombinant tissue plasminogen activator (rtPA) and one case with large vessel PSV was submitted to endovascular procedure (stenting).

In the Stroke sub-study, follow-up data regarding CVE recurrence data were available for 101 patients, and 12 (11.9%) had a recurrent CVE at 6-months follow-up: 6 (5.9%) stroke and 6 (5.9%) TIA. Regarding stroke disability, as measured by the mRankin score, data were available for 94 patients: 11 (11.7%) were dependent (score higher than 3), and five patients had died (mRankin 6) one due to 6-month CVE, 3 due to infection, and one of an unknown cause.

### Most clinical features of ischaemic CVE were comparable between patients with and without PSV

An analysis was conducted to explore whether features of CVE in the PSV DCVAS cohort (*n* = 97) differed from those present in patients without PSV from the DCVAS ‘PSV mimics’ cohort (*n* = 45) (Fig. 1). ‘PSV mimics’ included a heterogenous group of disorders, from other neurological and rheumatological conditions to infectious diseases (Online Resource 4).

Cardiovascular risk factors, stroke locations, disability and treatment were not different between groups. ‘PSV mimics’ patients were younger (median 48.0 [IQR 21.0] vs 61.0 [IQR 34.0] years, *p* = 0.02), less affected by malignancy (2.2% vs 13.7%, *p* = 0.03), and had lower frequencies of chest (15.6% vs 35.8%, *p* = 0.01), ear–nose–throat (13.3% vs 33.7%, *p* = 0.008), and gynaecological/urological (4.4% vs 17.9%, *p* = 0.02) involvement than those with PSV. Patients with PSV mimics had a higher frequency of CVE due to cardioembolism than patients with PSV (8.9% vs 1.0%, *p* = 0.04). Six-month CVE cumulative incidence was also similar (PSV mimics 6.8% vs PSV 11.9% *p* = 0.27), as well as disability (dependent PSV mimics 5.4% vs dependent PSV 11.7%, *p* = 0.23).

## Discussion

This study, based on data from the DCVAS cohort, has shown that CVE occurs in about 3.5% of patients with PSV at time of diagnosis. Rates of CVE varied among the different types of PSVs. Being an uncommon presentation, CVE may be challenging to diagnose and manage in patients with PSV. This study identified features that may help clinicians recognize CVE risk in PSV and showed that CVE in these patients is associated with significant disability and short-term recurrence.

Frequency of CVE varied according to PSV type. The highest frequencies were seen in Behçet’s and PAN, where previous limited data was available [40]. Interestingly, in Behçet’s disease, arterial ischaemic stroke was more common than CVT, a well-recognized neurological manifestation of this PSV [31], highlighting that these patients are also at risk of arterial ischaemic stroke as suggested before [41]. Regarding large vessel vasculitis, frequency of CVEs (3.6%) in GCA is similar to previous studies looking at stroke at the time of diagnosis [42, 43]. However, the TAK cohort had a lower frequency (6.0%) of CVE than what was previously reported [44], which could be explained by demographic differences, as the majority of the cases of TAK included in the DCVAS study were Asian, where lower rates of stroke have been described (1.2–1.3%) [44]. In the small-vessel vasculitis cohort CVE was less common, despite an expected higher risk of cardiovascular disease in this population [22, 24]. Cerebral small-vessel vasculitis may be more challenging to diagnose than the territorial infarcts associated with the large-vessel vasculitis, especially if detailed imaging (i.e. brain MRI) is not available. In the DCVAS EGPA cohort, CVE frequency (2.9%) was not much different compared to GCA, highlighting that the variability of CVE frequency across the different PSV is not explained by vessel type involvement alone, and should be recognized in all types of vasculitis.

The different types of PSV appear to have different predisposing factors for CVE occurrence. Overall and in patients with TAK, PAN, or Behçet’s disease, traditional vascular risk factors, such as smoking, arterial hypertension, diabetes, and dyslipidaemia, did not associate with the presence of CVE. On the other hand, a higher burden of vasculitis-related damage did associate with higher CVE risk, suggesting that CVE may be directly related to the vasculitic process. Additionally, in the small-vessel vasculitis group, although arterial hypertension associated with CVE, so did CRP, a marker of inflammation shown to be associated with cerebral small-vessel disease in MPO-ANCA-associated vasculitis at onset [9], again linking inflammation to cerebrovascular disease in these patients. Malignancy associated with a higher risk of CVE in our PSV patients and this association was not explained by the presence of concomitant vascular comorbidities, and the mechanisms of this association deserve to be further explored, especially in small-vessel vasculitis not associated with ANCA. We have confirmed and association between eye symptoms and CVE in the whole PSV cohort and this was likely driven by TAK and Behçet’s, highlighting the importance of ophthalmological assessment in patients with CVE and possible vasculitis [45].

Regarding the clinical features of stroke, ischaemic events were more frequent than haemorrhagic events, as expected. Large-vessel vasculitis associated with ischaemic territorial infarcts: in GCA most commonly affecting the posterior circulations and in TAK the anterior circulation, as previously described [42, 46, 47]. Recognizing these specific patterns of cerebrovascular involvement may be of help in differentiating GCA from TAK [47]. Stroke in small-vessel vasculitis presented with more heterogeneous features, frequently involving multiple vascular territories. During case review, aetiology was also more difficult to ascertain in these cases when compared to large-vessel vasculitis, because there was no obvious stenosis/occlusion or vascular wall enhancement as clear markers of a vasculitis-related mechanism of stroke. Again, these features may explain why it might be harder to recognize and classify stroke in these patients.

Finally, although direct vascular involvement is usually the easy go-to aetiology when assessing a PSV patient with stroke, our results also highlight that myocarditis/myocardiopathy needs to be considered as potential causes of ischemic stroke/TIA in PSV.

In exploratory comparisons, except for more widespread organ involvement, patients with PSV are not much different from the heterogenous group of the patients with mimics of PSV. Both PSV and PSV mimics have been reported to be more common in young patients with stroke than in the older population [4, 48].

The current study measured a 12% 6-month cumulative incidence of recurrent CVE, despite most patients being on at least one antiaggregant. Although this rate was not significantly different compared to PSV mimics (7%), this may be due to low sample size. We report a higher rate of recurrent events in PSV at 6 months than the rate reported at 12 months (3–6%) in a large study including patients of all ages [49] and of that reported at 5-years (9.4%) in young adults [50].

This study has several limitations. To seize the opportunity of studying a large group of patients with PSV, the Stroke substudy design was added to the main DCVAS study, to collect minimal additional information, and some design limitations could not be overcome (i.e. retrospective nature, time of scheduled visits). Because it was a large world-wide multicentre study, it was not possible to guarantee a standardized evaluation of CVE among all centres (i.e. previous medication; NIHSS not always available). However, this likely reflects real-world clinical practice where stroke work up is not uniform across centres [4]. To overcome this bias, all data on DCVAS study and Stroke CRF were reviewed by two neurologists to confirm the aetiologies of the CVEs. However, the investigators could not review the original imaging, and concomitant possible silent strokes or microvascular leukoencephalopathy could not be thoroughly assessed. Regarding the exploratory comparison with patients with CVE without vasculitis, the ‘PSV mimics’ group included a highly heterogenous group of diseases, which may halter direct comparisons. However, this is the main group of differential diagnosis of stroke associated with raised inflammatory markers or systemic involvement, and reflects clinical practice.

Despite its limitations, this study provides a comprehensive and unique insight into CVE in PSV. Its multicentric nature allowed the inclusion of a large number of patients with PSV and the estimation of frequencies of CVE. It also focuses on occurrence of CVE from symptom onset until diagnosis, compared to most previous studies that included patients only after diagnosis of PSV. This is important because stroke may be one of the first manifestations of PSV and understanding its clinical features may contribute to an early diagnosis of PSV. Moreover, CVEs at early stages of PSV are less likely to reflect side effects of long-term treatments such as glucocorticoid-related diabetes mellitus or infection. Though CVEs seem to be more frequent in PSV patients than in the general population, mainly occurring within the first years of PSV diagnosis [10, 51], lifelong stroke risk in PSV is not yet well-studied in prospective studies.

In conclusion, this study describes the prevalence and type of CVEs among patients with PSV. CVEs affect a minority of patients with PSV, but the frequency varies widely among different vasculitis. CVE in PSV is not explained by traditional vascular risk factors and has a moderate risk of short-term recurrence. These results have important implications to help identify patients with PSV who present with CVE and provide insight into the pathophysiology of CVEs in PSV.

### Supplementary Information

Below is the link to the electronic supplementary material.

Supplementary file1 (DOCX 25 KB)

Supplementary file2 (DOCX 24 KB)

Supplementary file3 (DOCX 22 KB)

Supplementary file4 (DOCX 22 KB)

Supplementary file5 (DOCX 22 KB)

Supplementary file6 (DOCX 1551 KB)
