# Supplementary material for: Stroke frequency, associated factors, and clinical features in primary systemic vasculitis: a multicentric observational study
Source: J Neurol. 2024 Mar 12;271(6):3309–20. doi: 10.1007/s00415-024-12251-1 (PMC11136713; doi:10.1007/s00415-024-12251-1)
Supplement: Supplementary file 1 — Supplementary file1 (DOCX 25 KB) [file 415_2024_12251_MOESM1_ESM.docx]

**Appendix 2: List of co-investigators for the DCVAS study**

| **Name** | **Location** | **Role** | **Contribution** |
| --- | --- | --- | --- |
| Paul Gatenby | ANU Medical Centre, Canberra, Australia | Site Investigator | Coordinated site data collection, including imaging |
| Catherine Hill | Central Adelaide Local Health Network: The Queen Elizabeth Hospital, Australia | Site Investigator | Coordinated site data collection, including imaging |
| Dwarakanathan Ranganathan | Royal Brisbane and Women's Hospital, Australia | Site Investigator | Coordinated centre site collection, including imaging |
| Andreas Kronbichler | Medical University Innsbruck, Austria | Site Investigator | Coordinated site data collection, including imaging |
| Daniel Blockmans | University Hospitals Leuven, Belgium | Site Investigator | Coordinated site data collection, including imaging |
| Simon Carette | Mount Sinai Hospital, Toronto, Canada | Site Investigator | Coordinated site data collection, including imaging |
| Navjot Dhindsa | University of Manitoba, Winnipeg, Canada | Site Investigator | Coordinated site data collection, including imaging |
| Aurore Fifi-Mah | University of Calgary, Alberta, Canada | Site Investigator | Coordinated site data collection, including imaging |
| Patrick Liang | Sherbrooke University Hospital Centre, Canada | Site Investigator | Coordinated site data collection, including imaging |
| Nataliya Milman | University of Ottawa, Canada | Site Investigator | Coordinated site data collection, including imaging |
| Christian Pineau | McGill University, Canada | Site Investigator | Coordinated site data collection, including imaging |
| Xinping Tian | Peking Union Medical College Hospital, Beijing, China | Site Investigator | Coordinated site data collection, including imaging |
| Guochun Wang | China-Japan Friendship Hospital, Beijing, China | Site Investigator | Coordinated site data collection, including imaging |
| Tian Wang | Anzhen Hospital, Capital Medical University, China | Site Investigator | Coordinated site data collection, including imaging |
| Ming-hui Zhao | Peking University First Hospital, China | Site Investigator | Coordinated site data collection, including imaging |
| Vladimir Tesar | General University Hospital, Prague, Czech Republic | Site Investigator | Coordinated site data collection, including imaging |
| Bo Baslund | University Hospital, Copenhagen (Rigshospitalet), denmark | Site Investigator | Coordinated site data collection, including imaging |
| Amira Shahin | Cairo University, Egypt | Site Investigator | Coordinated site data collection, including imaging |
| Laura Pirila | Turku University Hospital, Finland | Site Investigator | Coordinated site data collection, including imaging |
| Bernhard Hellmich | Kreiskliniken Esslingen, Germany | Site Investigator | Coordinated site data collection, including imaging |
| Julia Holle/ Frank Moosig | Klinikum Bad Bramstedt, Germany | Site Investigator | Coordinated site data collection, including imaging |
| Peter Lamprecht | University of Lübeck, Germany | Site Investigator | Coordinated site data collection, including imaging |
| Thomas Neumann | Universitätsklinikum Jena, Germany | Site Investigator | Coordinated site data collection, including imaging |
| Wolfgang Schmidt | Immanuel Krankenhaus Berlin, Germany | Site Investigator | Coordinated site data collection, including imaging |
| Cord Sunderkoetter | Universitätsklinikum Müenster, Germany | Site Investigator | Coordinated site data collection, including imaging |
| Zoltan Szekanecz | University of Debrecen Medical and Health Science Center, Hungary | Site Investigator | Coordinated site data collection, including imaging |
| Debashish Danda | Christian Medical College & Hospital, Vellore, India | Site Investigator | Coordinated site data collection, including imaging |
| Siddharth Das | Chatrapathi Shahuji Maharaj Medical Center, Lucknow (IP), India | Site Investigator | Coordinated site data collection, including imaging |
| Rajiva Gupta | Medanta, Delhi, India | Site Investigator | Coordinated site data collection, including imaging |
| Aman Sharma | Postgraduate Institute of Medical Education and Research, Chandigarh, India | Site Investigator | Coordinated site data collection, including imaging |
| Shrikant Wagh | Jehangir Clinical Development Centre, Pune (IP), India | Site Investigator | Coordinated site data collection, including imaging |
| Michael Clarkson | Cork University Hospital, Ireland | Site Investigator | Coordinated site data collection, including imaging |
| Eamonn Molloy | St. Vincent's University Hospital, Dublin, Ireland | Site Investigator | Coordinated site data collection, including imaging |
| Carlo Salvarani | Santa Maria Nuova Hospital, Reggio Emilia, Italy | Site Investigator | Coordinated site data collection, including imaging |
| Franco Schiavon | L'Azienda Ospedaliera of University of Padua, Itlay | Site Investigator | Coordinated site data collection, including imaging |
| Enrico Tombetti | Università Vita-Salute San Raffaele Milano, Italy | Site Investigator | Coordinated site data collection, including imaging |
| Augusto Vaglio | University of Parma, Italy | Site Investigator | Coordinated site data collection, including imaging |
| Koichi Amano | Saitama Medical University, Japan | Site Investigator | Coordinated site data collection, including imaging |
| Yoshihiro Arimura | Kyorin University Hospital, Japan | Site Investigator | Coordinated site data collection, including imaging |
| Hiroaki Dobashi | Kagawa University Hospital, Japan | Site Investigator | Coordinated site data collection, including imaging |
| Shouichi Fujimoto | Miyazaki University Hospital (HUB), Japan | Site Investigator | Coordinated site data collection, including imaging |
| Masayoshi Harigai/Fumio Hirano | Tokyo Medical and Dental University Hospital, Japan | Site Investigator | Coordinated site data collection, including imaging |
| Junichi Hirahashi | University Tokyo Hospital, Japan | Site Investigator | Coordinated site data collection, including imaging |
| Sakae Honma | Toho University Hospital, Japan | Site Investigator | Coordinated site data collection, including imaging |
| Tamihiro Kawakami | St. Marianna University Hospital Dermatology, Japan | Site Investigator | Coordinated site data collection, including imaging |
| Shigeto Kobayashi | Juntendo University Koshigaya Hospital, Japan | Site Investigator | Coordinated site data collection, including imaging |
| Hirofumi Makino | Okayama University Hospital, Japan | Site Investigator | Coordinated site data collection, including imaging |
| Kazuo Matsui | Kameda Medical Centre, Kamogawa< Japan | Site Investigator | Coordinated site data collection, including imaging |
| Eri Muso | Kitano Hospital, Japan | Site Investigator | Coordinated site data collection, including imaging |
| Kazuo Suzuki/Kei Ikeda | Chiba University Hospital, Japan | Site Investigator | Coordinated site data collection, including imaging |
| Tsutomu Takeuchi | Keio University Hospital, Japan | Site Investigator | Coordinated site data collection, including imaging |
| Tatsuo Tsukamoto | Kyoto University Hospital, Japan | Site Investigator | Coordinated site data collection, including imaging |
| Shunya Uchida | Teikyo University Hospital, Japan | Site Investigator | Coordinated site data collection, including imaging |
| Takashi Wada | Kanazawa University Hospital, Japan | Site Investigator | Coordinated site data collection, including imaging |
| Hidehiro Yamada | St. Marianna University Hospital Internal Medicine, Japan | Site Investigator | Coordinated site data collection, including imaging |
| Kunihiro Yamagata | Tsukuba University Hospital, Japan | Site Investigator | Coordinated site data collection, including imaging |
| Wako Yumura | IUHW Hospital (Jichi Medical University Hospital), Japan | Site Investigator | Coordinated site data collection, including imaging |
| Kan Sow Lai | Penang General Hospital, Malaysia | Site Investigator | Coordinated site data collection, including imaging |
| Luis Felipe Flores-Suarez | Instituto Nacional de Enfermedades Respiratorias, Mexico City, Mexico | Site Investigator | Coordinated site data collection, including imaging |
| Andrea Hinojosa-Azaola | Instituto Nacional de Ciencias Médicas y Nutrición Salvador Zubirán, Mexico City, Mexico | Site Investigator | Coordinated site data collection, including imaging |
| Bram Rutgers | University Hospital Groningen, Netherlands | Site Investigator | Coordinated site data collection, including imaging |
| Paul-Peter Tak | Academic Medical Centre, University of Amsterdam, Netherlands | Site Investigator | Coordinated site data collection, including imaging |
| Rebecca Grainger | Wellington, Otago, New Zealand | Site Investigator | Coordinated site data collection, including imaging |
| Vicki Quincey | Waikato District Health Board, New Zealand | Site Investigator | Coordinated site data collection, including imaging |
| Lisa Stamp | University of Otago, Christchurch, New Zealand | Site Investigator | Coordinated site data collection, including imaging |
| Emilio Besada | Tromsø, Northern Norway | Site Investigator | Coordinated site data collection, including imaging |
| Andreas Diamantopoulos | Hospital of Southern Norway, Kristiansand, Norway | Site Investigator | Coordinated site data collection, including imaging |
| Jan Sznajd | University of Jagiellonian, Poland | Site Investigator | Coordinated site data collection, including imaging |
| Elsa Azevedo | Centro Hospitalar de São João, Porto, Portugal | Site Investigator | Coordinated site data collection, including imaging |
| Miguel Rodrigues | Hospital Garcia de Orta, Almada, Portugal | Site investigator | Coordinated site data collection, including imaging |
| Yeong-Wook Song | Seoul National University Hospital, Republic of Korea | Site Investigator | Coordinated site data collection, including imaging |
| Sergey Moiseev | First Moscow State Medical University, Russia | Site Investigator | Coordinated site data collection, including imaging |
| Maria Cinta Cid | Hospital Clinic de Barcelona, Spain | Site Investigator | Coordinated site data collection, including imaging |
| Xavier Solanich Moreno | Hospital de Bellvitge-Idibell, Spain | Site Investigator | Coordinated site data collection, including imaging |
| Inoshi Atukorala | University of Colombo, Sri Lanka | Site Investigator | Coordinated site data collection, including imaging |
| Ewa Berglin | Umeå University Hospital, Sweden | Site Investigator | Coordinated site data collection, including imaging |
| Aladdin Mohammed | Lund-Malmo University, Sweden | Site Investigator | Coordinated site data collection, including imaging |
| Mårten Segelmark | Linköping University, Sweden | Site Investigator | Coordinated site data collection, including imaging |
| Haner Direskeneli | Marmara University Medical School, Turkey | Site Investigator | Coordinated site data collection, including imaging |
| Gulen Hatemi | Istanbul University, Cerrahpasa Medical School, Turkey | Site Investigator | Coordinated site data collection, including imaging |
| Sevil Kamali | Istanbul University, Istanbul Medical School, Turkey | Site Investigator | Coordinated site data collection, including imaging |
| Seval Pehlevan | Fatih University Medical Faculty ,Turkey | Site Investigator | Coordinated site data collection, including imaging |
| Matthew Adler | Frimley Health NHS Foundation Trust, Wexham Park Hospital, United Kingdom | Site Investigator | Coordinated site data collection, including imaging |
| Neil Basu | NHS Grampian, Aberdeen Royal Infirmary, United Kingdom | Site Investigator | Coordinated site data collection, including imaging |
| Iain Bruce | Manchester University Hospitals NHS Foundation Trust, United Kingdom | Site Investigator | Coordinated site data collection, including imaging |
| Kuntal Chakravarty | Barking, Havering and Redbridge University Hospitals NHS Trust, United Kingdom | Site Investigator | Coordinated site data collection, including imaging |
| Bhaskar Dasgupta | Southend University Hospital NHS Foundation Trust, United Kingdom | Site Investigator | Coordinated site data collection, including imaging |
| Oliver Flossmann | Royal Berkshire NHS Foundation Trust, United Kingdom | Site Investigator | Coordinated site data collection, including imaging |
| Nagui Gendi | Basildon and Thurrock University Hospitals NHS Foundation Trust, United Kingdom | Site Investigator | Coordinated site data collection, including imaging |
| Alaa Hassan | North Cumbria University Hospitals, United Kingdom | Site Investigator | Coordinated site data collection, including imaging |
| Rachel Hoyles | Oxford University Hospitals NHS Foundation Trust, United Kingdom | Site Investigator | Coordinated site data collection, including imaging |
| David Jayne | Cambridge University Hospitals NHS Foundation Trust, United Kingdom | Site Investigator | Coordinated site data collection, including imaging |
| Colin Jones | York Teaching Hospitals NHS Foundation Trust, United Kingdom | Site Investigator | Coordinated site data collection, including imaging |
| Rainer Klocke | The Dudley Group NHS Foundation Trust, United Kingdom | Site Investigator | Coordinated site data collection, including imaging |
| Peter Lanyon | Nottingham University Hospitals NHS Trust, United Kingdom | Site Investigator | Coordinated site data collection, including imaging |
| Cathy Laversuch | Taunton & Somerset NHS Foundation Trust, Musgrove Park Hospital, United Kingdom | Site Investigator | Coordinated site data collection, including imaging |
| Malgorzata Magliano | Buckinghamshire Healthcare NHS Trust, United Kingdom | Site Investigator | Coordinated site data collection, including imaging |
| Justin Mason | Imperial College Healthcare NHS Trust, United Kingdom | Site Investigator | Coordinated site data collection, including imaging |
| Win Win Maw | Mid Essex Hospital Services NHS Trust, United Kingdom | Site Investigator | Coordinated site data collection, including imaging |
| Iain McInnes | NHS Greater Glasgow & Clyde, Gartnavel Hospital & GRI, United Kingdom | Site Investigator | Coordinated site data collection, including imaging |
| John Mclaren | NHS Fife, Whyteman's Brae Hospital, United Kingdom | Site Investigator | Coordinated site data collection, including imaging |
| Matthew Morgan | University Hospitals Birmingham NHS Foundation Trust, Queen Elizabeth Hospital, United Kingdom | Site Investigator | Coordinated site data collection, including imaging |
| Ann Morgan | Leeds Teaching Hospitals NHS Trust, United Kingdom | Site Investigator | Coordinated site data collection, including imaging |
| Chetan Mukhtyar | Norfolk and Norwich University Hospitals NHS Foundation Trust, United Kingdom | Site Investigator | Coordinated site data collection, including imaging |
| Edmond O'Riordan | Salford Royal NHS Foundation Trust, United Kingdom | Site Investigator | Coordinated site data collection, including imaging |
| Sanjeev Patel | Epsom and St Helier University Hospitals NHS Trust, United Kingdom | Site Investigator | Coordinated site data collection, including imaging |
| Adrian Peall | Wye Valley NHS Trust, Hereford County Hospital, United Kingdom | Site Investigator | Coordinated site data collection, including imaging |
| Srinivasan Venkatachalam | The Royal Wolverhampton NHS Trust, United Kingdom | Site Investigator | Coordinated site data collection, including imaging |
| Erin Vermaak / Ajit Menon | Staffordshire & Stoke on Trent Partnership NHS Trust, Haywood Hospital, United Kingdom | Site Investigator | Coordinated site data collection, including imaging |
| Chee-Seng Yee | Doncaster and Bassetlaw Hospitals NHS Foundation Trust, United Kingdom | Site Investigator | Coordinated site data collection, including imaging |
| Daniel Albert | Dartmouth-Hichcock Medical Center, United States | Site Investigator | Coordinated site data collection, including imaging |
| Leonard Calabrese | Cleveland Clinic Foundation, United States | Site Investigator | Coordinated site data collection, including imaging |
| Sharon Chung | University of California, San Francisco, United States | Site Investigator | Coordinated site data collection, including imaging |
| Lindsy Forbess | Cedars-Sinai Medical Center, United States | Site Investigator | Coordinated site data collection, including imaging |
| Angelo Gaffo | University of Alabama at Birmingham, United States | Site Investigator | Coordinated site data collection, including imaging |
| Ora Gewurz-Singer | University of Michigan, United States | Site Investigator | Coordinated site data collection, including imaging |
| Kimberly Liang | University of Pittsburgh, United States | Site Investigator | Coordinated site data collection, including imaging |
| Eric Matteson | Mayo Clinic, United States | Site Investigator | Coordinated site data collection, including imaging |
| Rennie Rhee  Antoine Sreih | University of Pennsylvania, United States | Site Investigator | Coordinated site data collection, including imaging |
| Antoine Sreih | Rush University Medical Center, United States | Site Investigator | Coordinated site data collection, including imaging |
