# Supplementary material for: Stroke frequency, associated factors, and clinical features in primary systemic vasculitis: a multicentric observational study
Source: J Neurol. 2024 Mar 12;271(6):3309–20. doi: 10.1007/s00415-024-12251-1 (PMC11136713; doi:10.1007/s00415-024-12251-1)

**Title:** Stroke frequency, associated factors, and clinical features in primary systemic vasculitis: a multicentric observational study

**Authors:** Ruth Geraldes ^1,2^, Monica Santos ^3,4^, Cristina Ponte ^5,6^, Anthea Craven^7^, Lillian Barra^8^, Joanna C. Robson^9^, Nevin Hammam^10^, Jason Springer^11^, Jöerg Henes^12^, Alojzija Hocevar^13^, Jukka Putaala^14^, Ernestina Santos^15,16^, Liza Rajasekhar^17^, Thomas Daikeler^18^, Omer Karadag^19^, Andreia Costa^20^, Nader Khalidi^21^, Christian Pagnoux^22^, Patrícia Canhão^3,4^, Teresa Pinho e Melo^3,4^, Ana Catarina Fonseca^3,4^, José M Ferro ^23^, João Eurico Fonseca^5,6^, Ravi Suppiah ^24^, Richard A. Watts  ^25^, Peter Grayson ^26^, Peter A. Merkel ^27^ , Raashid A. Luqmani^7^

*On behalf of the DCVAS study group*

**Affiliations:**

1. Neurology department Wexham Park Hospital, Frimley Health Foundation Trust, Slough, UK
2. Department of Clinical Neurosciences, Oxford University Hospitals, Oxford, UK
3. Serviço de Neurologia, Departamento de Neurociências e Saúde Mental, Centro Hospitalar Universitário Lisboa Norte, Lisbon, Portugal
4. Centro de Estudos Egas Moniz, Faculdade de Medicina da Universidade de Lisboa, Lisbon, Portugal.
5. Rheumatology and Metabolic Bone Diseases Department, Centro Hospitalar Universitário Lisboa Norte, Lisbon Academic Medical Centre, Lisbon, Portugal
6. Rheumatology Research Unit, Instituto de Medicina Molecular, Faculdade de Medicina da Universidade de Lisboa, Lisbon Academic Medical Centre, Lisbon, Portugal.
7. Nuffield Department of Orthopaedics, Rheumatology and Musculoskeletal Sciences, Botnar Research Centre, Nuffield Orthopaedic Centre, University of Oxford, UK
8. Lawson Health Research Institute, London, Ontario, Canada
9. Centre for Health and Clinical Research, University of the West of England, Bristol, UK
10. Rheumatology Department, Faculty of Medicine, Assiut University, Assiut, Egypt.
11. University of Kansas Medical Centre Institute, USA
12. Centre for Interdisciplinary Clinical Immunology, Rheumatology and Auto-inflammatory Diseases and Department of Internal Medicine II (Oncology, Haematology, Immunology and Rheumatology), University Hospital Tuebingen, Germany
13. University Medical Centre Ljubljana, Slovenia
14. Helsinki University Central Hospital, Finland
15. Hospital de Santo António, Centro Hospitalar Universitário do Porto, Portugal
16. Unit for Multidisciplinary Research in Biomedicine, Instituto de Ciências Biomédicas de Abel Salazar, Universidade do Porto, Portugal
17. NIMS, Hyderabad, India
18. Department of Rheumatology and Department of Clinical Research, University Hospital, Basel, Switzerland
19. Division of Rheumatology, Department of Internal Medicine, Vasculitis Research Center, Hacettepe University School of Medicine, Ankara, Turkey
20. Centro Hospitalar Universitário de São João, Porto, Portugal e Neuroscience and Mental Health Department, Faculdade de Medicina da Universidade do Porto, Porto, Portugal
21. St Joseph’s Healthcare Hamilton and McMaster University, Ontario, Canada
22. Mount Sinai Hospital, Toronto, Canada
23. Instituto de Medicina Molecular, Faculdade de Medicina da Universidade de Lisboa, Lisbon, Portugal
24. Te Whatu Ora – Health New Zealand
25. Norwich Medical School, United Kingdom
26. National Institutes of Health, NIAMS Vasculitis Translational Research Program, Bethesda, USA
27. Division of Rheumatology, Department of Medicine, Division of Epidemiology, Department of Biostatistics, Epidemiology, and Informatics, University of Pennsylvania, Philadelphia, USA

Corresponding author: ruth.geraldes@ndcn.ox.ac.uk

**
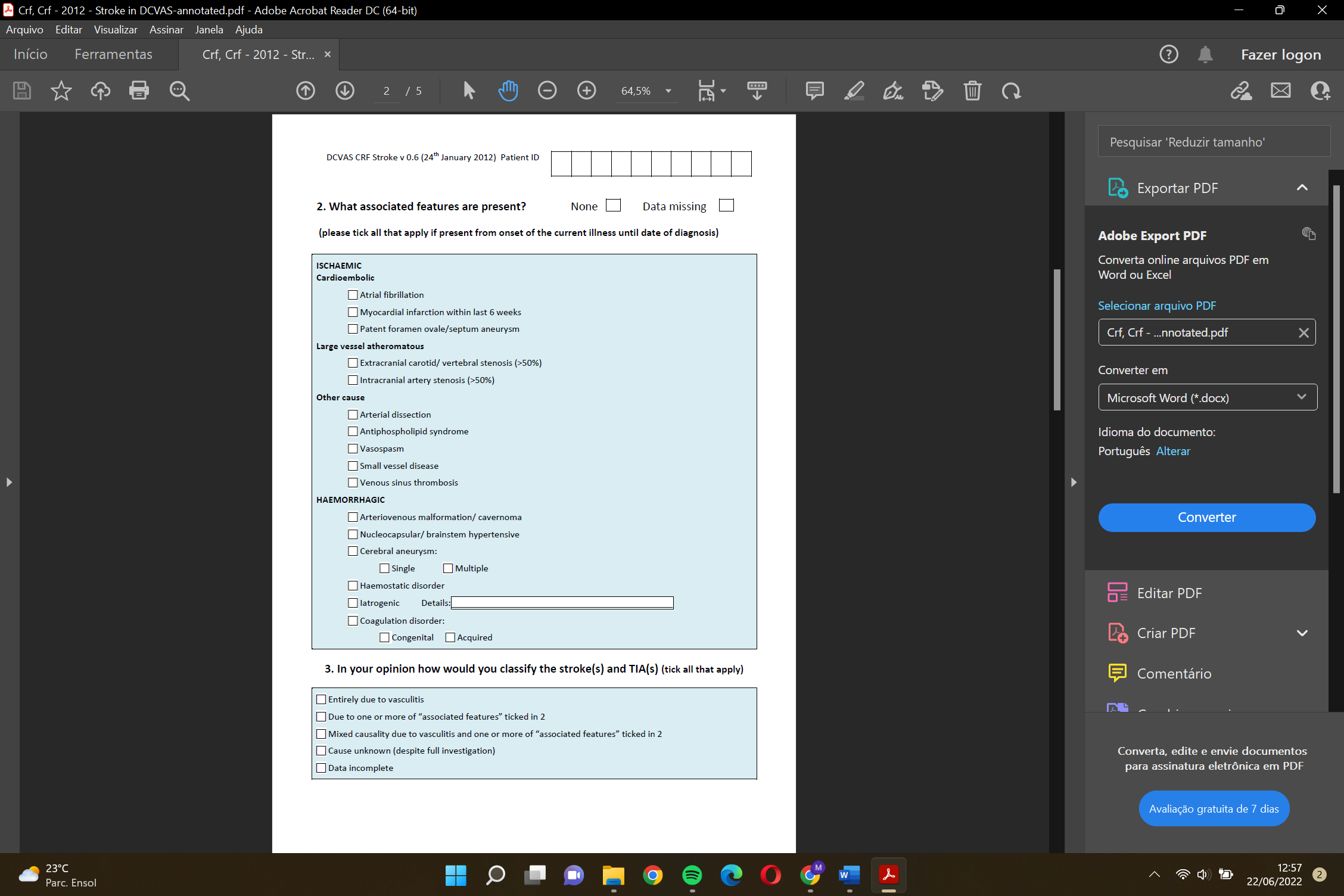

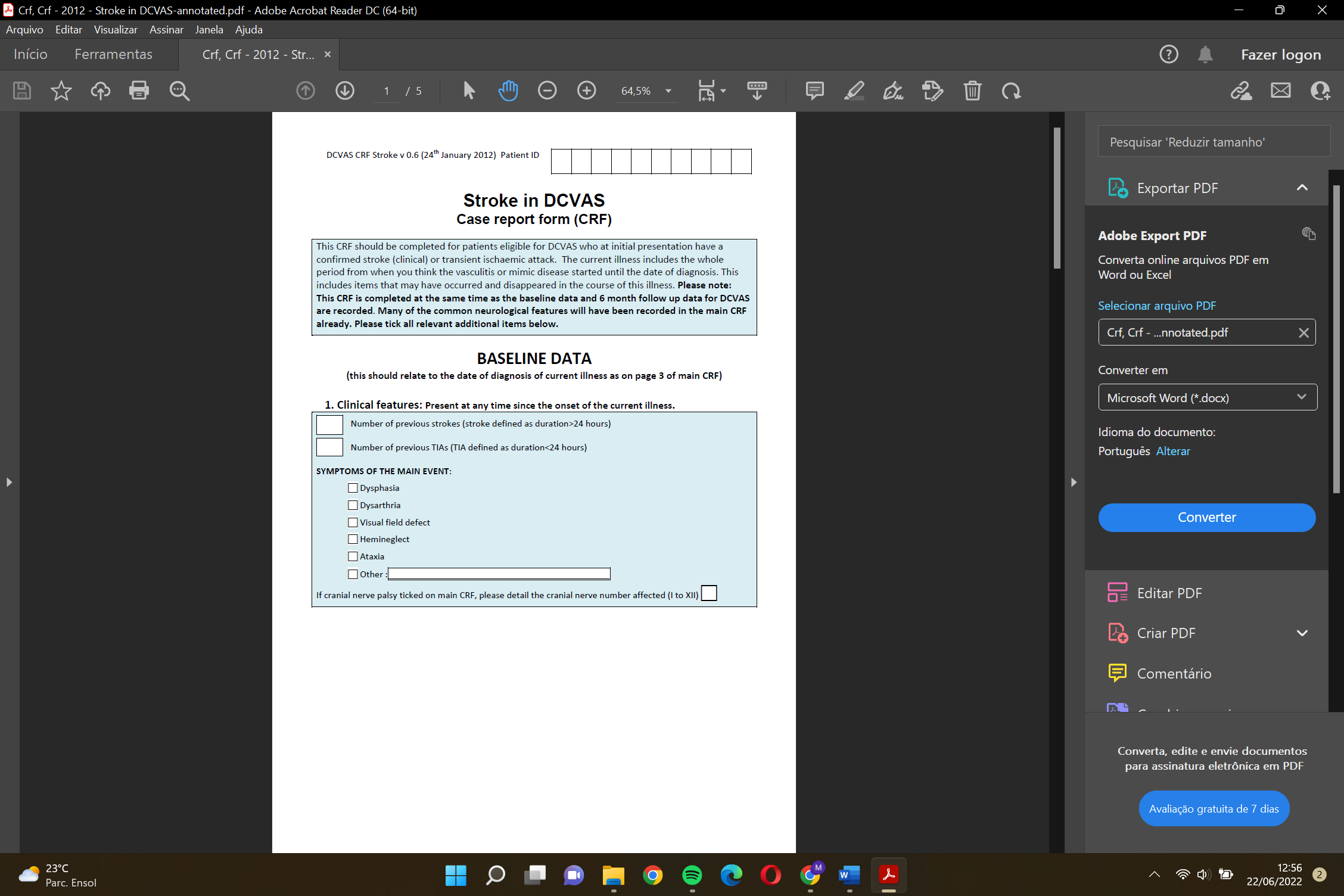
Online Resource 5. DCVAS Stroke Dataform**


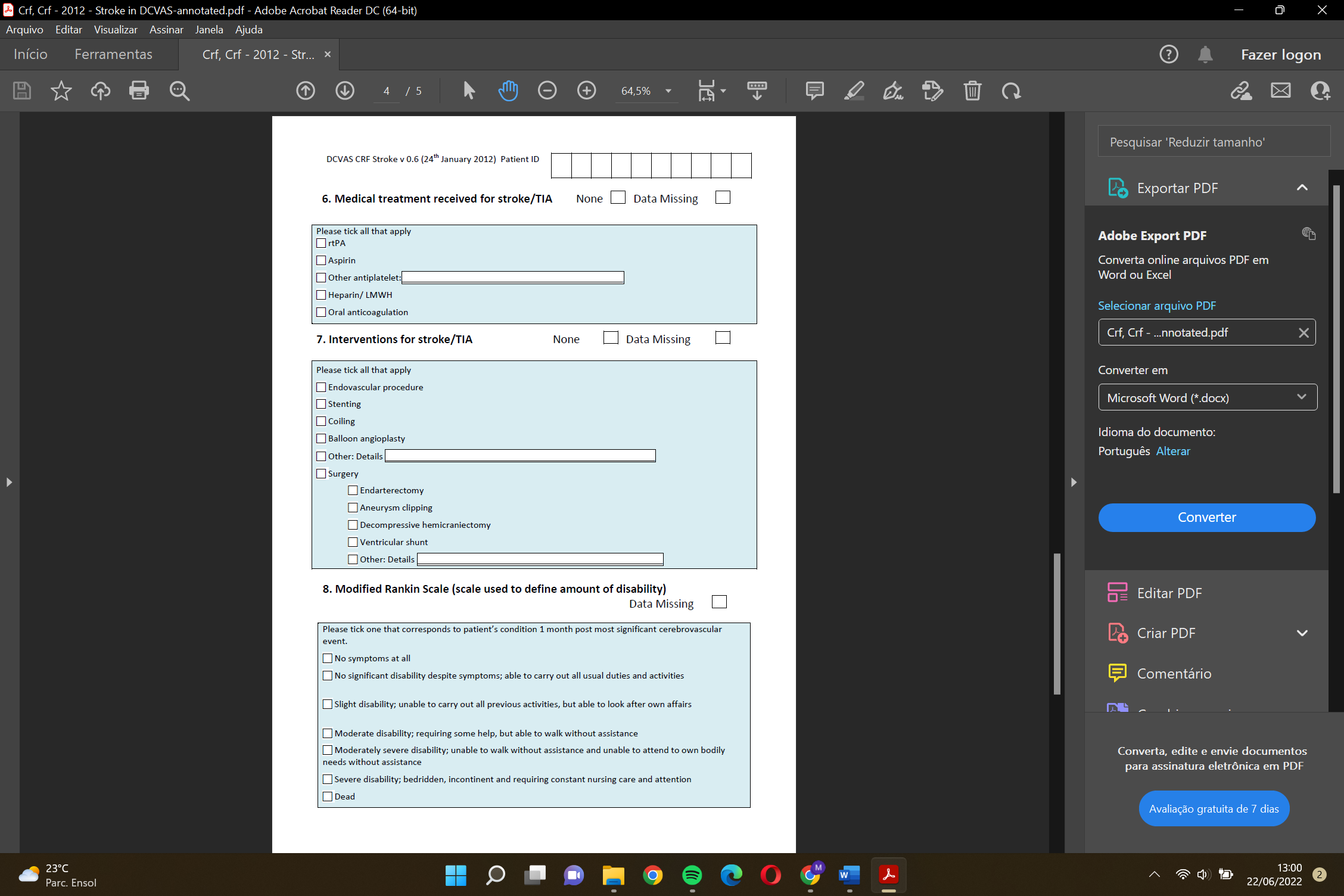

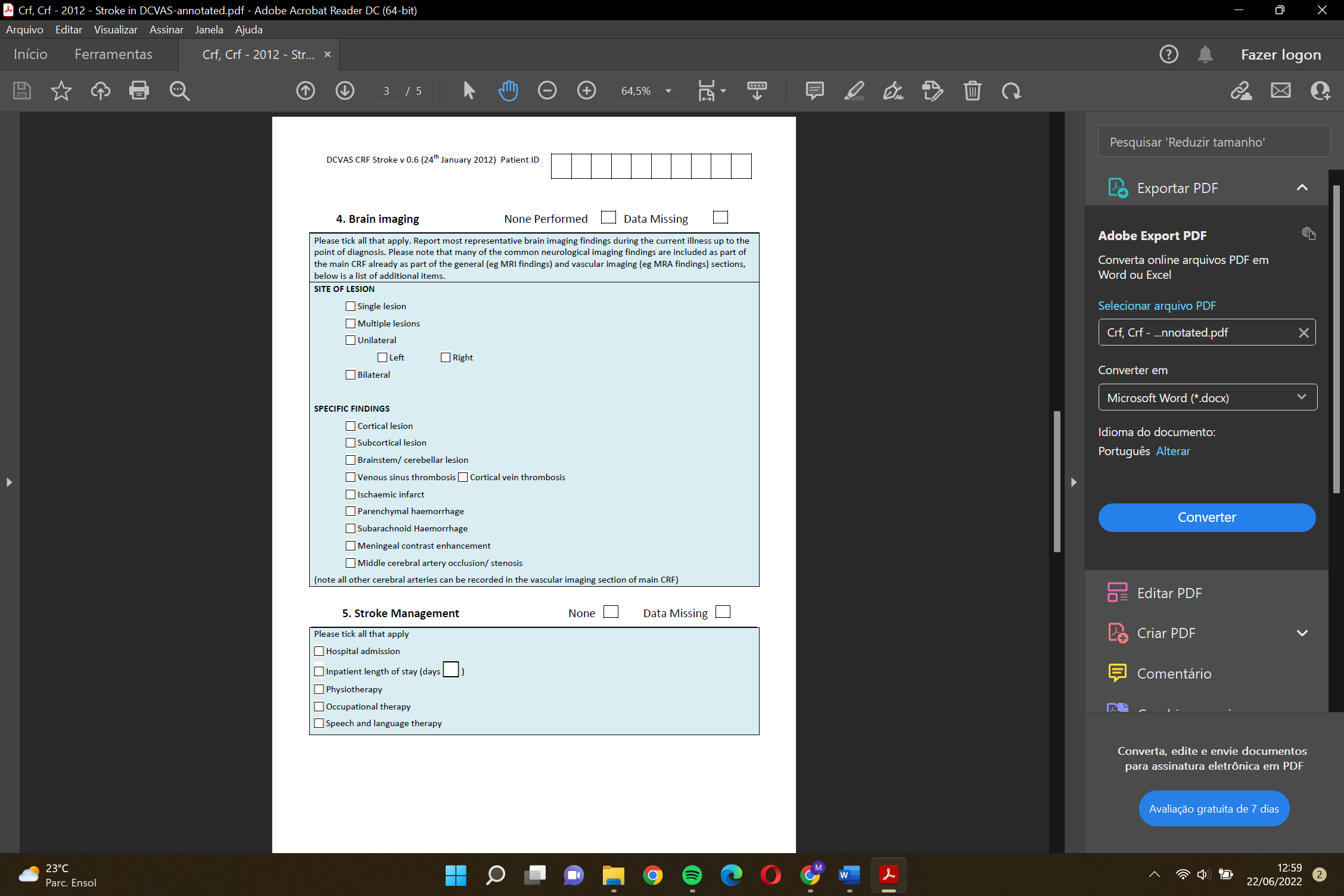


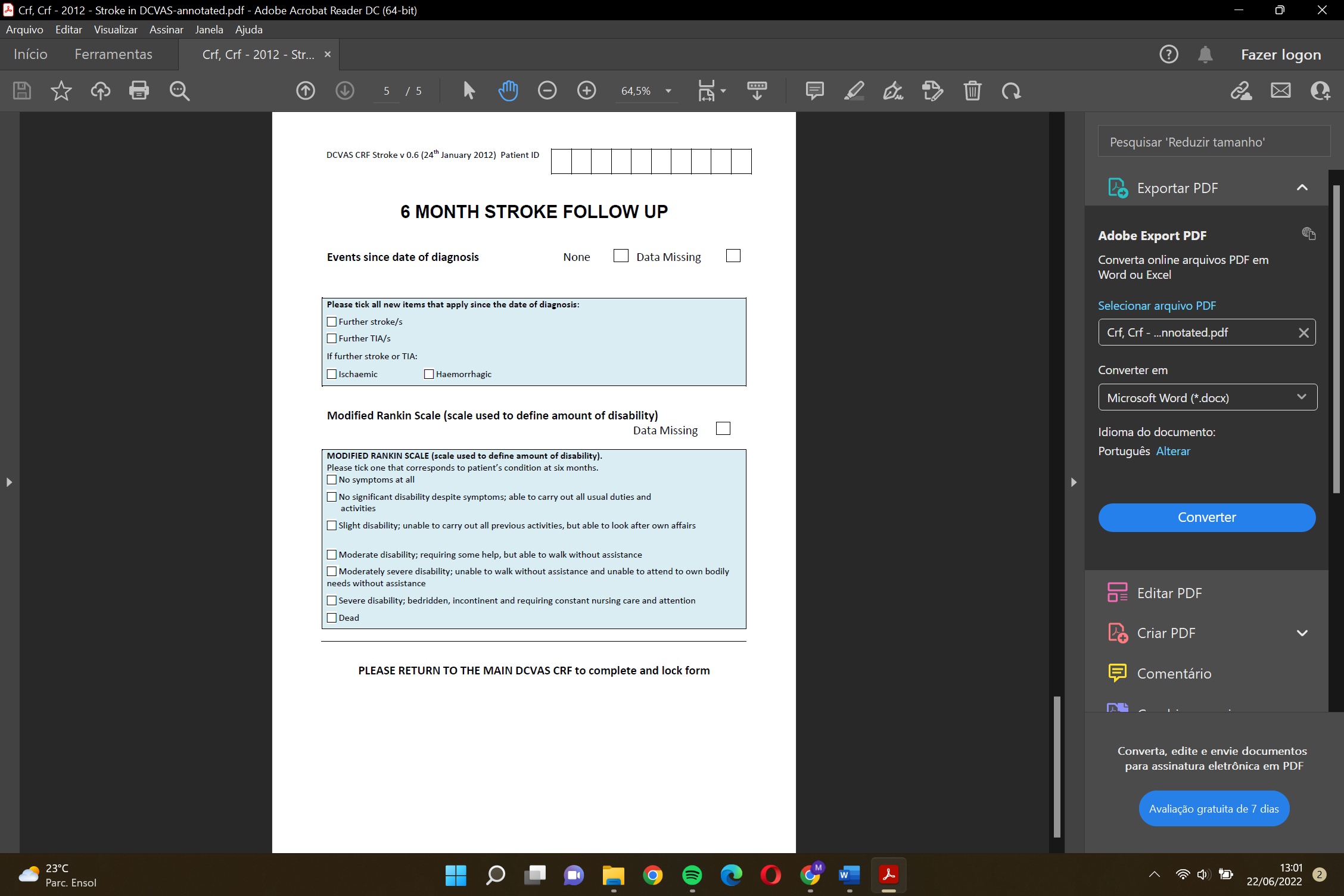

Supplement: Supplementary file 6 — Supplementary file6 (DOCX 1551 KB) [file 415_2024_12251_MOESM6_ESM.docx]
